# The Lepidoptera of White Sands National Monument, Otero County, New Mexico, USA 2. Rediscovery and description of *Sparkia immacula* (Grote, 1883) (Noctuidae, Noctuinae, Hadenini)

**DOI:** 10.3897/zookeys.149.1516

**Published:** 2011-11-24

**Authors:** Eric H. Metzler, Gregory S. Forbes

**Affiliations:** 1 Adjunct Curator of Lepidoptera, Michigan State University, P.O. Box 45, Alamogordo, NM 88311–0045 U.S.A.; 2 1009 Luna St, Las Cruces, New Mexico 88001 U.S.A.

**Keywords:** *Sparkia immacula*, Tularosa Basin, biological diversity, white gypsum dunes, Noctuidae, White Sands National Monument, New Mexico, National Park, Otero County, Arizona

## Abstract

In 2006 the U.S. National Park Service initiated a long term study of the Lepidoptera at White Sands National Monument, Otero County, New Mexico. *Sparkia immacula* (Grote, 1883), previously known only from historical specimens collected in Arizona and New Mexico, was discovered in the Monument in 2007 during the second year of the study. The adult moths and male and female genitalia are illustrated for the first time.

## Introduction

In 2006 the U.S. National Park Service invited the author to initiate a long-term study of the Lepidoptera at White Sands National Monument, Otero County, New Mexico. A primary purpose of the study was to compile an inventory of moths in habitats within and immediately adjacent to the white gypsum sand dunes in the Monument.

White Sands National Monument preserves 285 km^2^ (110 mi^2^), or about 40%, of the world’s largest snow-white gypsum dune field. The remainder of the 712 km^2^ (275 mi^2^) dune field is under the jurisdiction of the U.S. Army in the White Sands Missile Range. The dune field is located in the northern Chihuahuan Desert in southern New Mexico’s Tularosa Basin (Schneider-Hector 1993). A complete description of the study site and some of its unique biological resources is in Metzler et al. (2009).

There is a dearth of research on the invertebrate fauna in the gypsum dune field in the Tularosa Basin of New Mexico. Details of previous research pertinent to insects is given in Metzler et al. (2009).

In the period extending from 9 February 2007 through 31 December 2010 we identified more than 430 species of Lepidoptera (Metzler et al. unpublished data) from the Monument. This is the fourth in a series of papers pertinent to a detailed study of the Lepidoptera at White Sands National Monument (Metzler et al. 2009, Metzler et al. 2010a, Metzler et al. 2010b).

## Materials and methods

More than 250 samples of moths and other night flying insects were collected on 75 different nights in U.S.D.A. type black light traps, and at a black light or a mercury vapor light and sheet, as described in Covell (1984). A detailed description of the study methods is given in Metzler et al. (2009).

Genitalia were examined following procedures outlined in Clarke (1941), Hardwick (1950), Lafontaine (2004), and Pogue (2002). Abdomens were removed from the moths, wetted in 95% ethyl alcohol, and, using a dry bath, they were soaked in 10% KOH for 1.5 hours at 50°C. Genitalia were dissected in 5% ethyl alcohol, stained with Safranin O in 95% ethyl alcohol and Chlorazol Black E in water, dehydrated in 100% ethyl alcohol, cleared in clove oil, rinsed in xylene, and slide mounted in Canada balsam.

Terminology for elements of wing pattern, morphology, and genital structures follows Forbes (1954), Lafontaine (2004), and Mikkola et al. (2009). Forewing lengths were measured to the nearest ½ mm, from the base to the apex excluding fringe, using a stereo-microscope.

All specimens collected as part of a long-term study of Lepidoptera at White Sands National Monument are deposited in the following collections:

EHM personal collection of Eric H. Metzler, Alamogordo, New Mexico, USA, for subsequent transfer to MSU

MSU Albert J. Cook Arthropod Research Collection, Department of Entomology, Michigan State University, East Lansing, Michigan, USA

NMSU New Mexico State University Arthropod Collection, Las Cruces, New Mexico, USA

UNM Museum of Southwestern Biology, University of New Mexico, Albuquerque, New Mexico, USA

WHSA White Sands National Monument, New Mexico, USA

## Results

### Sparkia immacula

(Grote, 1883)

http://species-id.net/wiki/Sparkia_immacula

Figs 1, 2 5, 6 7

Cea immacula Grote 1883b: 78
Sparkia immacula ; Nye 1975; Franclemont 1983.

#### Type material.

*Sparkia immacula* was described from “Arizona.” The male holotype is deposited in the U.S. National Museum, Washington, DC (USNM). The abdomen of the holotype is missing. Another short series of *Sparkia immacula*, from Arizona, in the USNM, contains one male, also missing its abdomen.

#### Diagnosis.

**Figures 1–4.**
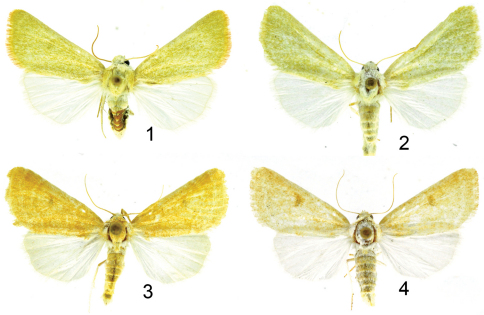
**1** *Sparkia immacula* (Grote) male **2** *Sparkia immacula* (Grote) female **3** *Trichocosmia inornata* Grote male **4** *Trichocosmia inornata* 0 female.

*Sparkia immacula* (figs 1–2) is a pale greenish-yellow noctuid moth without normal transverse markings or spots. The diagnostic features are 1) the pale yellowish-green color, and 2) lack of normal transverse markings and spots. *Sparkia immacula* might be mistaken for a species of *Schinia* Hübner, but *Schinia* have spines on the foretibia which are lacking in *Sparkia immacula*. *Sparkia immacula* flies with and is the same size as *Trichocosmia inornata* Grote 1883a (figs 3–4); *Trichocosmia inornata* is pale tan-ochre with faint transverse markings and a faint reniform spot. The frons is slightly rounded out.

#### Description.

**Figures 5–6.**
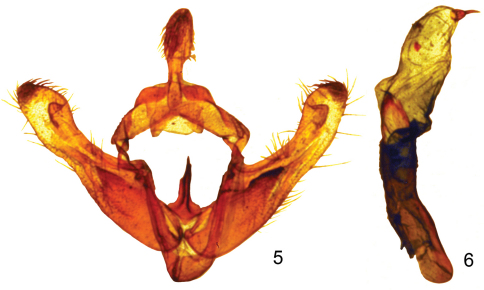
**5** *Sparkia immacula* male genitalia capsule **6** *Sparkia immacula* male genitalia aedeagus.

 Adult male (fig. 1). Head - front bulging, closely scaled, pale yellow and white; vertex scales narrow strap-like, erect, pale yellow and white; labial palpus white and pale yellow, erect, scales strap-like, closely scaled laterally and mesally, longer scales form longer fringe ventrally and shorter fringe dorsally. Haustellum coiled between labial palpi. Antenna filiform, dorsally pale yellow, closely scaled, ventrally setose, naked, brown. Thorax - dorsum pale yellow, scales long hair-like or strap-like; underside white, scales erect long hair-like. Legs pale yellow, closely scaled with long hair-like scales on ventral surface forming a shaggy fringe. Forewing: Length 12.5 mm (variation: 11.4–13.5 mm, mean 12.7 mm, n = 17.) Pale greenish yellow, transverse lines and spots typical of noctuines are absent; fringe pale yellow; underside pale greenish yellow; fringe pale greenish yellow. Hindwing white; fringe white; underside white; fringe white. Abdomen **-** dorsum closely scaled, whitish; underside whitish, closely scaled. Genitalia (fig. 5): tegumen expanded laterally with lateral lobes, uncus short, setose, spoon shaped; saccus short, U shaped, apex rounded; juxta with high ridge pointed anteriorly; valve tear drop shaped, costa sclerotized, ventral margin membranous, editum sclerotized, larger on right valve than left valve, digitus a sclerotized ridge in a thumb-like projection, sacculus sclerotized, well developed, cucullus moderately developed, corona with many mesally directed setae. Aedeagus (fig. 6) sclerotized, apex with blunt point, vesica membranous, straight, slightly shorter (.9×) than aedeagus, no diverticula, apex with strong cornutus.

**Figure 7.**
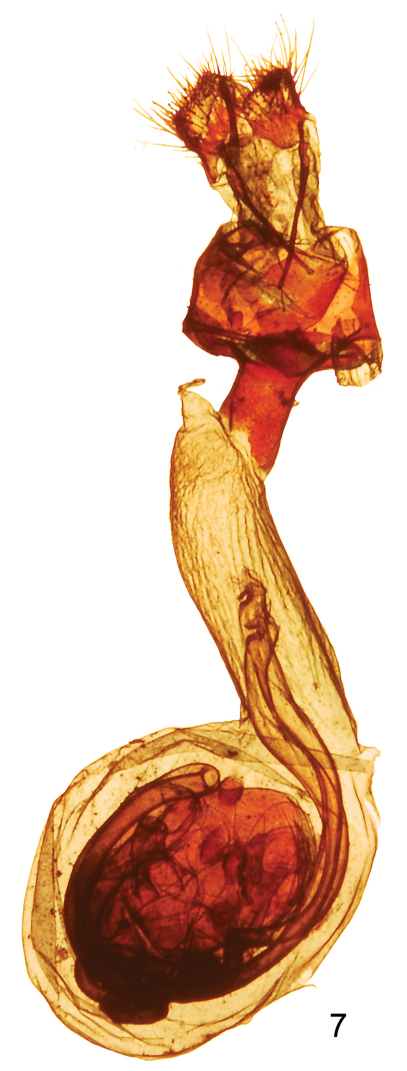
*Sparkia immacula* female genitalia.

Adult female (fig. 2): similar to male. Forewing length 12.5 - 14 mm, mean 13.1 mm, n = 9. Genitalia (fig. 7): Papilla analis not sclerotized, setose; posterior apophysis extend anteriorly to posterior margin of eighth segment; anterior apophysis length similar to posterior apophysis; ductus bursa sclerotized at posterior end, else membranous, elongate; corpus bursa oblong with four round signa.

#### Remarks.

*Sparkia immacula* is known from Arizona and New Mexico. Adult flight dates are 20 June through 25 August. The immature stages are unknown.

## Discussion

The study of Lepidoptera at White Sands National Monument is projected to last approximately 10 years.

## Supplementary Material

XML Treatment for Sparkia immacula
